# Patent ductus arteriosus ligation on neurodevelopmental outcomes at corrected 2 years

**DOI:** 10.1186/s13052-019-0698-7

**Published:** 2019-08-23

**Authors:** YoungAh Youn, Yu-mi Seo, Sook-Kyung Yum, In Kyung Sung

**Affiliations:** Department of Pediatrics, Seoul St. Mary’s Hospital, College of Medicine, The Catholic University of Korea, 222 Banpo-daero, Seocho-gu, 06591 Seoul, Republic of Korea

**Keywords:** Patent ductus arteriosus, Ligation, Outcome, Neurodevelopment

## Abstract

**Background:**

Our aim in this study was to evaluate whether very low birth weight infants (VLBWI) ligated for patent ductus arteriosus (PDA) were associated with worse neurodevelopmental outcomes at corrected 2 years. The ligated group was subdivided into ≤2 weeks of life (early) and ligated > 2 weeks of life (late) groups and compared the in-hospital morbidities and long term outcomes.

**Methods:**

Between Dec 2013 and Dec 2015, VLBWI diagnosed with hs PDA were evaluated.

**Results:**

Of the 191 VLBW infants with hs PDA, 28 (14.7%) infants had surgical ligation for PDA; 11 (39%) infants had EL and 17 (61%) infants had LL. Surgical ligation of hs PDA group had higher morbidities and mortality. Among the142 (83.0%) infants of 171 VLBWI with PDA survived, infants who were ligated had significantly lower scores of Bayley Scales of Infant and Toddler Development III at corrected age of 18 months. However, among the ligated group, there was little evidence of differences between the EL and LL groups. In a multivariable logistic regression analysis, only longer exposure of hs PDA and mechanical ventilation were consistently associated with worse neurodevelopmental outcomes.

**Conclusion:**

Our results suggest that surgical ligation for hs PDA may not increase risk for poor neurodevelopmental outcomes at corrected 2 years of age. The early surgical ligation may not be a risk factor.

## Strengths of this study

- Surgical ligation for hs PDA may not associated to increased risk for long-term outcomes at corrected 2 years
- Longer exposure of hs PDA and mechanical ventilation were consistently associated with worse neurodevelopmental outcomes.

## Limitations of our study

- Retrospective study design might not be the proper way to confirm examined relationships; selection bias may unintensionally occur
- Relatively small sample size of the study groups; especially, the small sample size in the ligated group may result in a lack of differences in outcomes

## Introduction

Patent ductus arteriosus (PDA) is one of the most frequently faced morbidities which accounts 70% of preterm infants born before 28 weeks’ gestation [1]. Its incidence is inversely related to gestational age, with an estimated incidence of up to as high as 80% for those born at 24–25 weeks gestation, depending on which diagnostic criteria is used [2]. Despite the higher incidence of PDA in more premature infants, we realized that examining hs (hemodynamically significant) PDA is important in assessing the clinical outcomes of very low birth weight infants (VLBWI). To standardize and to closely monitor hs PDA in VLBWI to avoid any adverse hospital outcomes, we applied the comprehensive McNamara and Sehgal criteria [3] of PDA to infants in our neonatal intensive care unit (NICU) beginning in 2012. This criterion is based on echocardiographic findings in combination with clinical findings and is staged from mild to severe Our target for treatment of hs PDA included infants with at least moderate-stage HSDA (stage C3) based on the criteria developed by McNamara and Sehgal [3]. Severe and prolonged exposure of hs PDA without any intervention in premature infants has been shown to increase morbidities, such as intraventricular hemorrhage (IVH), necrotizing enterocolitis (NEC), kidney injury, heart failure, and chronic lung disease (CLD) [4–8]. Furthermore, persistent hemodynamically significant left-to-right PDA shunts increased mortality. Therefore, after careful selection of hs PDA, surgical ligation of ductus arteriosus was usually considered when medical treatment (oral ibuprofen) has failed or is contraindicated. Meanwhile, surgery itself can be associated with significant short and long-term complications. The brain appears to be more susceptible to the neurotoxic effects of anesthesia during periods of rapid growth and development, as well as peak synaptogenesis [9]. Additionally, a large multicenter cohort study has shown that major surgery in VLBWI was independently associated with an increased risk of death or neurodevelopmental impairment at 18–22 months’ corrected age [10]. Our aim in this study was to evaluate whether VLBWI ligated were associated with worse neurodevelopmental outcome at corrected 18 months in addition to hospital morbidities and mortality. Further, the ligated group was subdivided into ≤2 weeks of life (early) and ligated > 2 weeks of life (late) groups and compared the short and long term outcomes.

## Materials and methods

Between Dec 2013 and Dec 2015, VLBWI with hs PDA admitted to our Neonatal Intensive Care Unit (NICU) in Seoul St. Mary’s Hospital were evaluated and followed up until 18 month after discharge. The clinical data for these infants relating to hs PDA, hospital morbidities, mortality and neurodevelopmental outcome were retrospectively collected for analysis. VLBWI were screened for PDA within the first 4 days of life, and their diagnoses were confirmed with a two-dimensional echocardiogram. If hs PDA was found in the first week of assessment, serial echocardiogram and close monitoring were continued weekly until hs PDA was resolved. Persistent hs PDA was treated either pharmacologically with oral ibuprofen or with surgical ligation for VLBWI who were assessed to be at least moderate-stage hs PDA (stage C3) based on the criteria developed by McNamara and Sehgal.^3^ The VLBWI with complex congenital heart disease or right-to-left or bidirectional PDA shunting were excluded from this study. No infant received prophylactic treatment for PDA. Throughout the study, oral ibuprofen was prescribed at 10 mg/kg of ibuprofen as an initial dose, followed by two additional doses of 5 mg/kg on consecutive days [11, 12].

Surgical ligation of PDA was performed on VLBWI who could not be treated pharmacologically due to various clinical contraindications (e.g., acute renal failure (ARF), positive DIC with bleeding tendency, etc.) or because pharmacological interventions failed. Surgical ligation of PDA was routinely performed at the patient’s bedside in the NICU, and all ligations were performed by one experienced pediatric thoracic and cardiovascular surgeon using a metal clip through the third or fourth intercostal space after posterolateral thoracotomy. The study was approved by the Ethics Committee of Seoul St. Mary’s Hospital, The Catholic University of Seoul, Korea.

### Definitions

By definition, surgical ligation of PDA ≤ 2 weeks was defined as early ligation (EL) and surgical ligation of PDA > 2 weeks was defined as late ligation (LL) in this study.

The hs PDA patients were those who meet at least moderate stage (stage ≥3) of clinical and echocardiographic criteria as proposed by McNamara and Sehgal [3]. Medical response was defined as a reduction of PDA size to less than 1.5 mm and improvement in both clinical and echocardiographic criteria (stage ≤2). Medical failure was defined as a persistent hs PDA (stage ≥3), with a ductal size greater than 1.5 mm following 2 courses of oral ibuprofen or any cases in which oral ibuprofen was unable to be used following the1st course, due to acute renal failure (ARF), necrotizing enterocolitis (NEC) or to positive DIC laboratory findings.

Resuscitation at birth was defined as oxygen use or positive pressure ventilation or intubation or cardiac massage or drug use. Pulmonary hypertension is defined by the need to use nitric oxide or sildenafil or iloprost ≤1 week of life. BPD was diagnosed if oxygen use exceeding 0.21% was still needed at a corrected gestational age of 36 weeks. NEC was defined as grade II or higher using Bell’s classification. Intraventricular hemorrhage (IVH) > grade II was defined as active bleeding in the ventricles, and the grade designation was based on Drs. Papile and Levene’s classification criteria. Retinopathy of prematurity (ROP) was defined as the classified according to the International Classification of Diseases for ROP. At corrected age of 18–24 months, VLBWI with PDA who survived completed the cognitive, language and motor composites of the Bayley Scales of Infant and Toddler Development III. Children were considered at risk if scores were > 2 SDs below the test mean (scores of < 70).

### Statistical analysis

Continuous variables were compared using Student’s t-test and are expressed as the means ± of the standard deviations. Discrete variables were compared using a χ2 test or Fisher’s exact test and are expressed as percentages. Missing data was extracted from the analysis. All of the analyses were two-tailed, and clinical significance was defined as a *p* value lower than 0.05. To seek any confounding risk factors for surgical ligation of PDA, we used a multivariate logistic regression model. Odds ratios (ORs) and 95% confidence intervals (CI) were calculated using a multivariate statistical model that included the following predictors related to surgical ligation of PDA with a stepwise logistic regression analysis: gestational age, birth weight, pulmonary hypertension, IVH > grade II and neonatal seizure. All the statistical analyses were performed with the Statistical Package for the Social Sciences (SPSS), version 15.0 (SPSS-PC Inc., Chicago, IL, USA).

## Results

### Demographic data

Of 507 VLBWI, 191 (37.6%) infants were diagnosed with hs PDA in our NICU of Seoul St. Mary’s Hospital, The Catholic University of Korea between December 2013 and December 2015. Of the 191 VLBW infants with hs PDA, 28 (14.7%) infants had surgical ligation for PDA; 11 (39%) infants had EL and 17 (61%) infants had LL. Regarding complications of surgical ligation, all surgically ligated patients did not have any reported serious complications associated with ligation as vocal cord paralysis [13]. The mean surgical time was an average duration of 21 min.

Among the VLBWI with PDA, the mean gestational age and birth weight were significantly lower in the PDA ligated group compared to non-ligated group (27.2 ± 12.7 vs 28.7 ± 17.8, 1003 ± 186 vs 1070 ± 285, *p* = 0.001), Additionally, pulmonary hypertension ≤1 week of life, oral ibuprofen use, exposure to hs PDA and neonatal seizures were significantly more prevalent in the ligated group (*p* < 0.05). As in morbidities, the rate of retinopathy of prematurity (ROP) laser treatment, BPD ≥ moderate, duration of mechanical ventilation and hospitalized days were significantly higher and longer in the ligated group (*p* < 0.05). The mortality was also significantly higher in the ligated group (Table 1).

**Table 1:** Clinical characteristics of VLBWIs (*n* = 191) with PDA

	No surgical ligation (*n* = 163)	Surgical ligation group (*n* = 28)	*P*-value
Gestational age, week	28.2 ± 17.8	27.2 ± 12.7	0.001
Birth weight, g	1070 ± 285	1003 ± 186	0.235
Male, n (%)	140 (85.9)	11(39.3)	0.338
Resuscitation at birth	155 (97.5)	23 (82.1)	0.058
RDS^b^	151 (92.6)	25 (92.6)	0.993
Pneumothorax^b^	21(12.9)	4 (14.3)	0.839
Pulmonary hemorrhage^b^	33 (20.2)	7 (25.0)	0.568
Pulmonary hypertension^a,b^	17 (10.4)	12(42.9)	< 0.001
Oral ibuprofen use^b^	9 (5.5)	10 (35.7)	< 0.001
Exposure to hs PDA,days^b^	7.2 ± 9.6	21.5 ± 24.6	< 0.001
IVH > grade II	17 (19.5)	14 (24.1)	0.053
Neonatal seizure^b^	14 (16.1)	20 (34.5)	0.010
Sepsis	60(36.8)	15 (53.6)	0.093
NEC operation	19 (12.0)	4 (14.8)	0.685
ROP laser Treatment	7/156(4.3)	4 (14.8)	0.036
BPD ≥ moderate	19 (21.8)	14 (50.0)	0.004
TPN, days	34.1 ± 28.6	61.5 ± 34.6	0.886
Mechanical ventilation, days^c^	20.3 ± 27.8	40.4 ± 38.5	0.026
Hospitalized days ^c^	54.7 ± 37.9	76.7 ± 41.2	< 0.001
PVL	64 (43.2)	8 (29.6)	0.186
Mortality	15(9.2)	5(17.8)	0.048

*Abbreviations*: *VLBWI* very low birth weight infants, *PDA* patent ductus arteriosus, *g* gram body weight, *RDS* respiratory distress syndrome, *IVH* intraventricular hemorrhage, *NEC* necrotizing enterocolitis, *ROP* retinopathy of prematurity, *BPD* bronchopulmonary dysplasia, *TPN* total parental nutrition, *PVL* periventricular leukomalacia

^a^Use of nitrioxide, sildenafil or iloprost within 1 week of birth

^b^Clinical variables before surgical ligation

^c^Expired patients were excluded

At corrected age of 2 years, 142 (83.0%) infants of 171 VLBWI with PDA survived and returned for follow -up evaluations and completed the cognitive, language and motor composites of the Bayley Scales of Infant and Toddler Development III. There were significant differences in cognitive, language and motor composites of the Bayley Scales between the PDA ligated and no ligated groups. The infants who had PDA ligation had significantly lower scores for the all three (cognitive, language and motor) composites of Bayley Scales of Infant and Toddler Development III (Table 2). In addition, the infants at high risk defined as in the method section were significantly higher in the PDA ligated group (Table 2). However, among the ligated group, there was little evidence of differences in cognitive, language, or motor composite scores between the EL and LL groups (Table 3).

**Table 2:** VLBWI with hs PDA Outcomes on Bayley Scales of Infant and Toddler Development III at corrected 18 months

	No surgical ligation (*n* = 121/148)	Surgical ligation group (*n* = 21/23)	*P*-value
Score, mean ± SD			
Cognitive	95.7 ± 15.1	83.9 ± 17.1	0.048
Language	92.5 ± 13.0	81.9 ± 19.8	0.025
Motor	86.7 ± 13.8	70.7 ± 13.6	0.036
At -risk, n (%)^a^			
Cognitive score	6 (5.0)	17 (81.0)	< 0.001
Language score	6 5.0)	17 (81.0)	< 0.001
Motor score	6 (5.0)	17 (81.0)	< 0.001
Catch up growth	91/101 (90.1)	17/21 (81.0)	0.231

^a^Children were considered at risk if scores were > 2 SDs below the test mean (scores of < 70)

**Table 3:** VLBWI who were ligated for PDA; Outcomes on Bayley Scales of Infant and Toddler Development III at corrected 18 months

	Early surgical ligation (*n* = 9)	Late Surgical ligation (*n* = 14)	*P*-value
Score, mean ± SD			
Cognitive	86.3 ± 11.1	82.0 ± 22.0	0.719
Language	88.5 ± 3.8	83.8 ± 27.7	0.726
Motor	76.0 ± 11.5	71.8 ± 24.4	0.763
At -risk, n (%)^a^			
Cognitive score	2(22.2)	4 (28.6)	0.735
Language score	2 (22.2)	4 (28.6)	0.735
Motor score	2 (22.2)	4 (28.6)	0.735
Catch up growth^a^	8 (88.9)	9 (64.2)	0.422

^a^Children were considered at risk if scores were > 2 SDs below the test mean (scores of < 70)

In order to find a consistent relationship between the neurodevelopmental outcomes and surgical ligation, we performed a multivariable logistic regression. We included gestational age, exposure to hs PDA, IVH > grade II, mechanical ventilation along with PDA ligation in this analysis. Only longer duration for exposure to hs PDA and mechanical ventilation duration were consistently associated with worse neurodevelopmental outcomes (Table 4).

**Table 4:** Effects on neurodevelopmental outcomes (adjusted for PDA ligation, gestational age, exposure to hs PDA, IVH > grade II, and mechanical ventilation) in a multiple logistic regression analysis

	*P*-value	OR	95% CI
Cognitive			
PDA ligation	0.656	1.424	0.301–6.747
Gestational age	0.060	0.961	0.678–1.363
Exposure to hs PDA^a^	0.021	8.598	1.389–5.237
IVH > grade II	0.133	2.362	0.769–7.255
Mechanical ventilation^a^	0.011	5.402	1.461–19.975
Language			
PDA ligation	0.980	0.985	0.326–3.229
Gestational age	0.083	2.270	0.898–5.741
Exposure to hs PDA^a^	0.038	5.85	1.104–3.080
IVH > grade II	0.236	1.844	0.670–5.074
Mechanical ventilation^a^	0.010	4.054	1.394–11.791
Motor			
PDA ligation	0.963	0.968	0.239–3.914
Gestational age	0.082	2.359	0.896–6.211
Exposure to hs PDA^a^	0.033	2.952	1.093–7.977
IVH > grade II	0.602	0.764	0.274–2.132
Mechanical ventilation^a^	0.026	2.805	1.128–6.976

*Abbreviations*: *PDA* patent ductus arteriosus, *hs* hemodynamically significant, *IVH* intraventricular hemorrhage

^a^Days of duration

## Discussion

The prevalence of hs PDA is inversely related to maturity. The hs PDA affects 60% of infants born weighing less than < 1000 g of extremely pre-term infants and our incidence of PDA in VLBWI was 37.6%, which was similar to the 30% reported incidence of PDA in VLBWI [1].

Our study manifested that surgical ligation of hs PDA group were not significantly associated with worse neurodevelopmental outcomes at 18 months follow-up after adjusting variable neonatal factors. We concluded that only longer exposure to hs PDA and more ventilator care were significantly associated with worse neurodevelopmental outcomes. Weisz et al. [14] manifested a similar result that retrospective cohort study PDA ligation among preterm babies was not associated with the composite outcome of death or neurodevelopmental impairment (NDI) compared with medically managed babies. He mentioned that bias associated with premature related complications may result as a confounding factor to worse clinical outcomes in VLBWI surgically ligated. In this study, the ligation was also not associated with worse neurodevelopmental outcomes. Additionally, Qureshi et al. [15] reported a multivariate analysis of a retrospective cohort of preterm infants with PDA, adjusting for PDA-related illness severity at the time of the decision to treat the PDA. Meta-analysis of these studies revealed no difference in the composite outcome of death or NDI between surgically and medically treated infants. Furthermore, Mohamed MA et al. [16] also revealed that conservative management (no treatment) of PDA may not compromise survival without CLD and is not associated with increased morbidities in VLBW infants. Accordingly, surgical ligation of hs PDA can be performed in selected infants with persistent hs PDA.Other than PDA ligation itself, the general anesthesia associated with surgery may have a negative influence on neurodevelopment of those VLBWI. A large multicenter cohort study has also recently shown that major surgery in VLBWI was independently associated with an increased risk of death or neurodevelopmental impairment at 18–22 months’ corrected age [10, 17]. Since PDA ligation can be performed shortly, our mean surgical time was an average duration of 21 min with no complications, it can be helpful in weaning ventilator and oxygen need in addition to shorten the hospital days in VLBWI. Among the ligated group, the EL and LL were not significantly different in morbidity, mortality and long term outcome at corrected 18 months. This may indicate the timing of ligation is not important, but the need to find hs PDA is crucial in the prognosis of VLBWI. When the hs PDA is persistent, management with initially oral ibuprofen can be tried and if medical failure was concluded, timed and selective surgical ligation can be performed even before ≤2 weeks of life in VLBWI. Prolonged hemodynamic and respiratory derangement due to diastolic steal phenomenon by large ductal shunting may result in longer mechanical ventilation period, subsequent BPD and other adverse hospital outcomes. In addition to BPD, necrotizing enterocolitis (NEC) is also frequently reported postnatal morbidities of pulmonary over-circulation due to the ductal “steal phenomenon [18–20].” Due to possibilities of these morbidities, 70% of those born before 28 weeks of gestation reported to receive either medical or surgical therapy to close the PDA [1]. In the future, studies using near infrared spectroscopy have raised new concerns about the potential for long-term neurodevelopmental problems following prolonged PDA exposure because of decreased cerebral oxygenation in the presence of a persistent PDA [21].

Additionally, hs PDA infants who were ligated had more incidence of BPD ≥ moderate in the ligated group. This may be due to longer exposure to pulmonary edema resulting from hs PDA which may be eventually resolved by surgical ligation. A recent study in premature baboons demonstrated altered pulmonary mechanics and arrested alveolarization after 14 days of exposure to moderate-sized ducti [22]. Over-circulation of the lungs in humans can result in microcirculatory lung damage [23]. Furthermore, previous study asserted that both clinical and radiographic pulmonary edema and lung compliance were improved after early surgical ligation [24] which may result early resolution of pulmonary edema by surgical ligation of hs PDA. The pulmonary hypertension at ≤1 week of life which was significantly more associated in LL group may be due to the low compliance with high resistant lungs within the first week of life which may further delay spontaneous closing of ductus arteriosus. Any, who could benefit from PDA treatment.

Because surgical ligation is an effective and definite procedure associated with low mortality, when indicated as hs PDA, timed and selective ligation without delay of the hs PDA exposure period for VLBWI may be considered. The optimal timing for surgical ligation should not depend merely on the size of the ductus but depend on the hs PDA which compromises major organs and may effect on the rate of morbidities and mortality. The surgical time for hs PDA ligation is any time when hs PDA is diagnosed and persisted even before 2 weeks of life in VLBWI. Otherwise, pulmonary over-circulation may lead to considerable morbidity involving BPD, NEC and renal impairment, due to the “steal phenomenon.” We are aware of some limitations of our study: (i) the retrospective study design might not be the proper way to confirm examined relationships; selection bias may unintensionally occur (ii) the relatively small sample size of the study groups; especially, the small sample size in the ligated group may result in a lack of differences in outcomes.(iii) many clinical conditions co-mingle originating from prematurity itself.

## Conclusions

Our results suggest that surgical ligation for hs PDA may not increase risk for poor neurodevelopmental outcomes at corrected 18 months follow up. Timing of surgical ligation of PDA may not be a risk factor for worse outcomes in selected hs PDA infants. Further prospective well designed studies eliminating bias are needed to determine the direct effect of PDA ligation on the neurodevelopmental outcomes of VLBWI.

## Data Availability

The datasets used during the current study are available from the corresponding author. Medical records are available in the Archive of the Department of Pediatrics of the Seoul St. Mary’s Hospital.

## Abbreviations

ARF: Acute renal failure
CLD: Chronic lung disease
EL: Early ligation
hs: hemodynamically significant
IVH: Intraventricular hemorrhage
LL: Late ligation
NEC: Necrotizing enterocolitis
PDA: Patent ductus arteriosus
PVL: Periventricular leukomalacia
ROP: Retinopathy of prematurity
VLBWI: Very low birth weight infants
